# Stability and individual variability of social attachment in imprinting

**DOI:** 10.1038/s41598-021-86989-3

**Published:** 2021-04-12

**Authors:** Bastien S. Lemaire, Daniele Rucco, Mathilde Josserand, Giorgio Vallortigara, Elisabetta Versace

**Affiliations:** 1 Center for Mind and Brain Sciences, University of Trento, Trento, Italy; 2 Department of Psychology, University of Milano-Bicocca, Milan, Italy; 3 Laboratory Dynamique du Language, University of Lyon 2, Lyon, France; 4 School of Biological and Chemical Sciences, Queen Mary University of London, London, UK

**Keywords:** Learning and memory, Social behaviour

## Abstract

Filial imprinting has become a model for understanding memory, learning and social behaviour in neonate animals. This mechanism allows the youngs of precocial bird species to learn the characteristics of conspicuous visual stimuli and display affiliative response to them. Although longer exposures to an object produce stronger preferences for it afterwards, this relation is not linear. Sometimes, chicks even prefer to approach novel rather than familiar objects. To date, little is known about how filial preferences develop across time. This study aimed to investigate filial preferences for familiar and novel imprinting objects over time. After hatching, chicks were individually placed in an arena where stimuli were displayed on two opposite screens. Using an automated setup, the duration of exposure and the type of stimuli were manipulated while the time spent at the imprinting stimulus was monitored across 6 days. We showed that prolonged exposure (3 days vs 1 day) to a stimulus produced robust filial imprinting preferences. Interestingly, with a shorter exposure (1 day), animals re-evaluated their filial preferences in functions of their spontaneous preferences and past experiences. Our study suggests that predispositions influence learning when the imprinting memories are not fully consolidated, driving animal preferences toward more predisposed stimuli.

## Introduction

Young social animals that move around soon after birth, such as ducklings and domestic chicks, require to stay in contact with conspecifics to survive and thrive^1^. Hence, it is not surprising that at the beginning of life, they can quickly learn the features of the mother and stay in contact with her, a phenomenon known as filial imprinting^2–8^. Imprinting has become a model for understanding memory, learning and the onset of social behaviour in neonate animals^1,9–12^. Imprinting responses are not only observed in the wild, where they are directed to the mother or siblings^13^. In laboratory settings, chicks imprint on objects^3^ such as plastic cylinders^14–16^ and computer monitor displays^17–19^. This paves the way for systematic studies in controlled laboratory conditions. As little as 15 min of visual exposure is sufficient for chicks to develop a learned preference for a conspicuous object^20^. Nonetheless, the strength of the preference varies depending on the imprinting object used. Chicks imprinted with a predisposed stimulus—a stimulus they spontaneously approach—show a higher preference than chicks imprinted with non-predisposed stimulus. These results suggest that filial preferences are influenced by experience (exposure to an object) and the animal’s predispositions. In this study, we investigate the interface of predispositions and imprinting when the exposure to an object is increased from several hours to a few days. Salzen and Meyer showed that chicks change their imprinting preferences toward a novel object after prolonged exposure to it^21^. In contrast, other studies^22,23^ showed irreversible imprinting if a predisposed stimulus (such as a live hen) is used as a primary imprinting stimulus, again suggesting a close relationship between filial preferences and predispositions.

It has been suggested that predispositions direct the chick’s attention toward the kind of stimuli from which the animal would benefit the most^24–26^. In fact, chicks have predisposed (not learned) preferences for patterns of motion^27–29^ and arrangments of features^30–32^ that are similar to those found in living animals, such as biological motion^33,34^, self-propulsion^35,36^ or even specific colours such as red (which is the colour of the comb, a zone of the head important for individual recognition^37^). Predispositions for patterns of motions and colours can affect the acquisition of imprinting memory^25^. Chicks exposed to biological motion (point-light displays of a moving hen) form a learned colour preference more effectively. Moreover, the association of predisposed features such as biological motion and red colour located on the chick’s head makes imprinting more robust^26^.

Colours are used to discriminate between individuals in a chicken flock^37^. In filial imprinting, the young animals use colour as an essential characteristic to recognise their imprinting objects^38^ and some colours appear more effective than others^3^. Although the effect of the contrast between a colour and its background has not been clarified yet, red, orange and blue appear to elicit stronger responses than green and yellow^39–42^. Therefore, red and blue can be considered as predisposed imprinting stimuli. In our study, we used objects of different colours to investigate whether spontaneous preferences are steady or can change in time.

Filial imprinting preferences have been well described^3,6^. However, how these preferences develop in time and vary depending on the animals’ predispositions has been poorly documented. We know that longer exposure (from a couple of minutes to a few hours) produces stronger preferences for the imprinting stimulus (familiar stimulus)^4,20,43^. However, after imprinting, the preference for approaching familiar objects and avoiding novel objects is not merely steady nor incremental. On the contrary, in some situations, chicks prefer to approach novel rather than familiar objects, an unexpected behaviour. For instance, Bateson et al.^20,44^ have observed that in the initial stage of imprinting—i.e. 15 and 30 min after the beginning of the imprinting phase but not after 60 min—chicks are motivated to be exposed to novel objects. More recently, the early shift from the first object to the exploration of alternative stimuli has been observed in different breeds of chicks that were tested on their spontaneous preferences to approach a stuffed hen versus a scrambled version of it. Versace et al. have shown that while in the first 5 min of visual experience, all breeds had a preference for the stuffed hen, 5 min later, one breed started to explore the other stimulus^45^. Interestingly, preferences for novel stimuli in imprinting appear also at much later stages^16,17^. In this paper, we focus on imprinting responses up to 6 days after hatching.

The longitudinal aspect of our study enables us to investigate the paradoxical phenomenon of the preference for unfamiliar imprinting objects. While it has been shown that exploration of novelty takes place at different stages of imprinting, how and why this counterintuitive phenomenon appears remains an open question. To date, the transient preference for unfamiliar stimuli, named ‘slight-novelty preference’ by Bateson, has been described and modelled as a phenomenon driven by the need to explore different points of view of the imprinting stimulus to build a full representation of it^46^. According to this hypothesis, the preference for exploring objects slightly different from the imprinting stimulus would help recognise different points of view of the mother hen and build a complete representation of it^6,44,47^. This hypothesis is supported by other studies showing that when two stimuli are presented in close temporality, they became “blended” as a unique stimulus for the animals^48,49^. However, the hypothesis that (only) in the first hour of exposure chicks explore novel stimuli to improve the imprinting object’s representation has been confuted by behavioural^16,17^ and physiological/biochemical studies^12,50–52^. For instance, a novelty preference has been observed after the first day of imprinting. After 3 days of imprinting, chicks prefer novel visual patterns presented as a sequence of stimuli^16^ or as simultaneous multimodal pattern^17^. Interestingly, sex differences have been observed^17^, with males preferring unfamiliar stimuli and females preferring familiar stimuli^53,54^.

Little is known about individual differences in imprinting behaviour. Templeton and Smith described that chicks’ response to an effective stimulus varied across a wide range of performance and was not affected by genetics^55^. Gribosvkiy et al.^56^ developed a quantitative methodology to study the inter-individual variability among chicks in imprinting and showed high variability between individuals and behavioural types. Chicks with higher behavioural flexibility had a stronger preference for novelty in a generalisation task after conditioning^57^. Little is known whether individual differences apply to imprinting, mainly due to the difficulties in tracking and analysing imprinting behaviour at the individual level across time. To overcome this difficulty, we use automated behavioural tracking techniques^58–61^, studying individual imprinting preferences for multiple consecutive days.

We built an automated setup to continuously track chicks’ behaviour from the first exposure to the imprinting stimuli for six consecutive days. Chicks were individually housed in an arena with two opposite monitors. The imprinting and test stimuli position was counterbalanced between monitors while we measured the distance of chicks from the stimuli. Imprinting duration and testing duration were manipulated. Objects of different colours were used as imprinting objects to investigate the role of colour on chicks preferences. In Experiment 1, chicks were imprinted for 1 day with one stimulus and tested for 5 days with two stimuli. In Experiment 2, the imprinting duration was increased to 3 days and chicks were tested for 3 days. In Experiment 3, chicks were imprinted with one object for 1 day, then with another one for 2 days and tested for 3 days. In Experiment 4, we replicated a similar procedure than Experiment 3, but this time assessed the animal preference between their primary or secondary imprinting object. In such settings, with prolonged and continuous behavioural monitoring, we investigated how filial preferences developed in time at the group and individual level.

## Results

### Experiment 1

#### Imprinting

There were non-significant effects of Condition (imprinted with a green hourglass or imprinted with a blue cube; *F*(1, 28) = 0.57, *p* = 0.46), Sex (*F*(1, 28) = 0.18, *p* = 0.67) or interaction Sex × Condition, *F*(1, 28) = 0.14, *p* = 0.71) on the time spent close to the imprinting stimulus. The chicks significantly remained closer to the imprinting stimulus (*t*(31) = 83.25, *p* < 0.001, Cohen’s *d* = 14.72), spending 96% of their time (± 0.56 SEM) close to the imprinting stimulus. All chicks (32) remained significantly more on the side of the arena in which the imprinting stimulus was displayed (Table S1 in the Supplementary Material).

#### Testing

**Figure 1:**
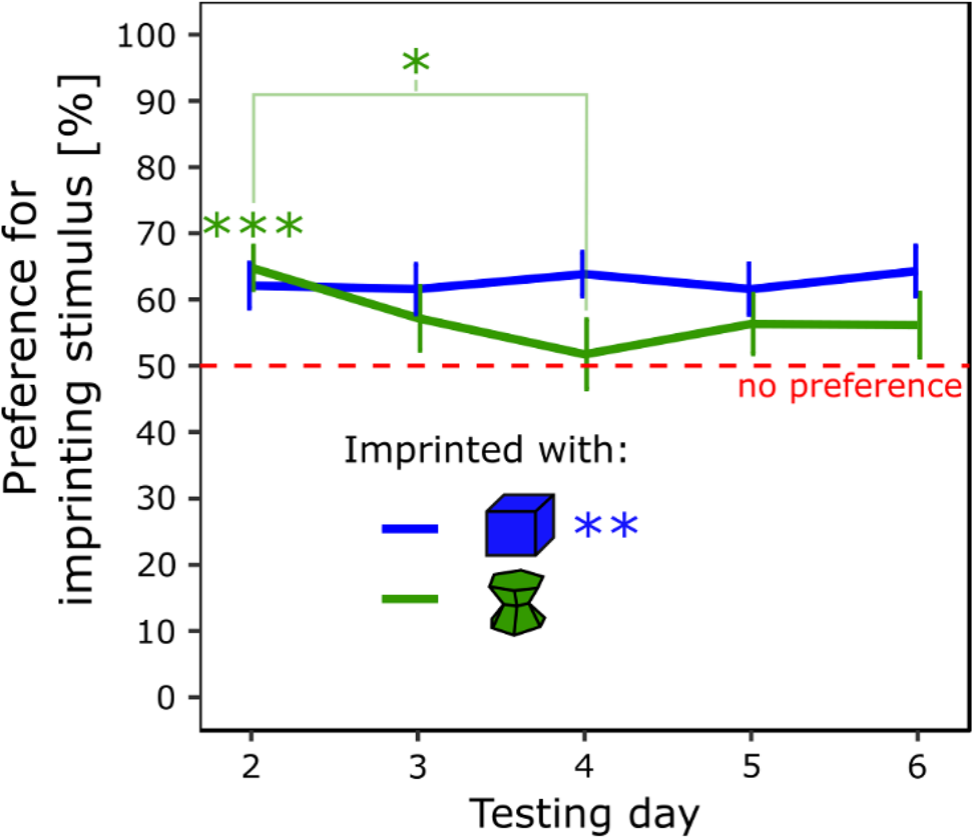
In experiment 1, the preference for the imprinting stimulus was stable stable across days for the chicks imprinted with the blue stimulus (blue line) but not with the green stimulus (green line) (p < 0.05, *; p < 0.01, **; p < 0.001, ***). Green asterisks represent the statistical significance of the group of chicks imprinted with the green stimulus. The blue asterisks represent the statistical significance of the group of chicks imprinted with the blue stimulus.

The results are shown in Fig. 1. There were non-significant effects of Condition (*F*(1, 28) = 0.89, *p* = 0.37), Sex (*F*(1, 28) = 0.50, *p* = 0.49), Day (*F*(4, 112) = 1.06, *p* = 0.38) or interactions (Sex × Condition *F*(1, 28) = 0.009, *p* = 0.93; Sex × Day, *F*(4, 112) = 0.28, *p* = 0.89; Sex × Condition × Day, *F*(4, 112) = 0.40, *p* = 0.81), but a significant interaction between Day and Condition on the Preference for the imprinting stimulus (*F*(4, 112) = 2.69, *p* < 0.05). Post hoc analysis (Tukey) showed that the preference for the imprinting stimulus observed on day 2 was significantly different from the preference observed on day 4 in the green condition (*t*(112) = 3.52, *p* < 0.05, Cohen’s *d* = 0.74). On day 2, chicks had a significant preference for the imprinting stimulus (*t*(15) = 4.45, *p* < 0.001, Cohen’s *d* = 1.12) and spent 65% (± 3.31 SEM) of their time close to it. However, on day 4, chicks had no preference (*t*(15) = 0.33, *p* = 0.75, Cohen’s *d* = 0.082) and spent 52% (± 5.26 SEM) of their time close to their imprinting stimulus. The post hoc test did not reveal other differences. Chicks imprinted with the blue stimulus had a significant and stable preference for the imprinting stimulus (*t*(15) = 3.83, *p* < 0.01, Cohen’s *d* = 0.96) and spent 62% (± 3.23 SEM) of their time close to it.

In the blue condition, 10 chicks (63%) had a significant preference for the imprinting stimulus, 5 (31%) had no preference, and 1 (6%) significantly preferred the unfamiliar stimulus. In the green condition, 7 chicks (44%) had a significant preference for the imprinting stimulus, 6 (37%) had no preference, and 3 (19%) had a significant preference for the unfamiliar stimulus (Table S1 in the Supplementary material). Levene’s test showed that the variances of the two conditions were similar (*F*(1, 30) = 0.32, *p* = 0.86).

### Experiment 2

#### Imprinting

There were non-significant effects of Condition (*F*(1, 28) = 1.15, *p* = 0.29), Sex (*F*(1, 28) = 0.002, *p* = 0.97) or interaction (Sex × Condition, *F*(1, 28) = 3.3, *p* = 0.08) on the time spent close to the imprinting stimulus. The trend revealed above was induced by an opposite pattern of between males and females within each condition with small variances. Nonetheless, the time spent close to the imprinting stimulus between each group was similar. Overall, the chicks significantly remained close the imprinting stimulus (*t*(31) = 49.92, *p* < 0.001, Cohen’s *d* = 8.82) 93% of their time (± 0.46 SEM). All chicks (32) chose significantly more the side of the arena, where the imprinting stimulus was displayed (Table S2 in the Supplementary Material).

#### Testing

**Figure 2:**
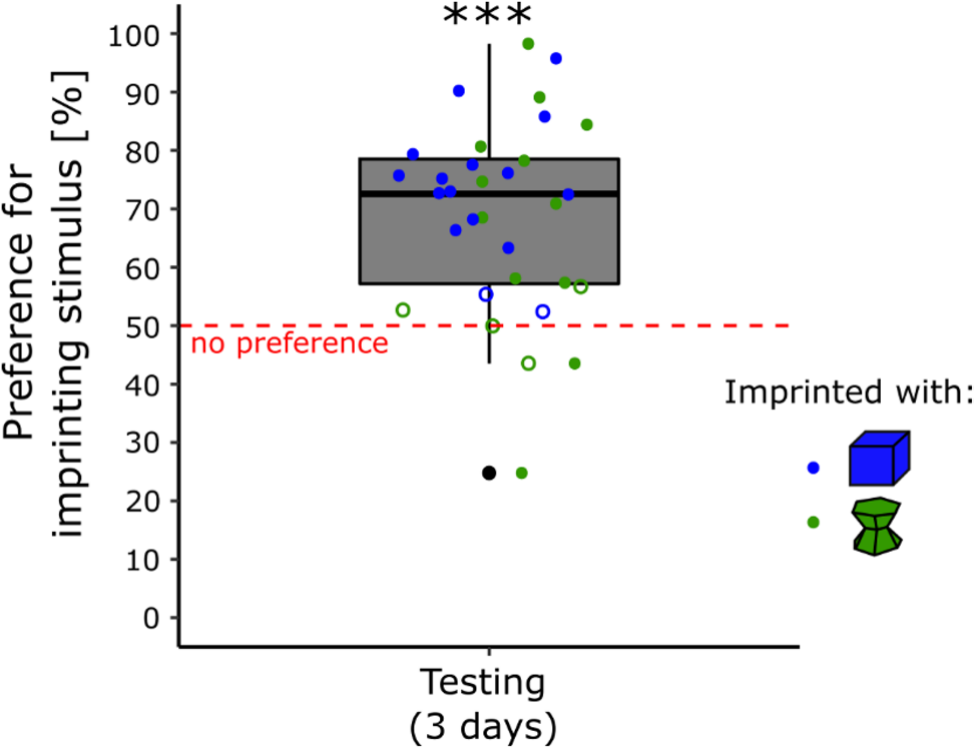
In experiment 2, the preference for the imprinting stimulus was stable across days for both conditions (p < 0.001, ***). The blue dots represent the preference score of the chicks imprinted with the blue stimulus. The green dots represent the preference score of the chicks imprinted with the green stimulus. Filled dots show the individuals having a significant preference while empty dots show the individuals having no preference. The asterisks represent the overall significance of both conditions pooled, against no preference.

The results are shown in Fig. 2. There were non-significant effects of Condition (F(1, 28) = 2.90, p = 0.10), Sex (F(1, 28) = 2.12, p = 0.16), Day (F(2, 56) = 0.63, p = 0.54) or interactions (Sex × Condition, *F*(1, 28) = 0.003, *p* = 1.0; Sex × Day, *F*(2, 56) = 0.05, *p* = 0.95, Condition × Day, *F*(2, 56) = 0.46, *p* = 0.63; Sex × Condition × Day, *F*(2, 56) = 1.52, *p* = 0.23) on the Preference for the imprinting stimulus. The preference for the imprinting stimulus was significantly different from chance-level (*t*(31) = 6.58, *p* < 0.001, Cohen’s *d* = 1.16). The chicks spent on average 69% (± 2.90 SEM) of their time close to their imprinting stimulus.

In the blue condition, 14 chicks (87.5%) had a significant preference for the imprinting stimulus, 2 (12.5%) had no preference, and none significantly preferred the unfamiliar stimulus. In the green condition, 10 chicks (62.5%) had a significant preference for the imprinting stimulus, 4 (25%) had no preference, and 2 (12.5%) had a significant preference for the unfamiliar stimulus (Table S2 in the Supplementary Material). Levene’s test showed that the variances of the two conditions were significantly different (*F*(1, 30) = 6.14, *p* < 0.05). Chicks imprinted with the green stimulus showed higher variability in their preferences for the imprinting stimulus during testing (σ^2^ = 380.85) than chicks imprinted with the blue stimulus (σ^2^ = 129.91).

### Experiment 3

#### Primary imprinting

There were non-significant effects of Condition (*F*(1, 29) = 0.52, *p* = 0.48), Sex (*F*(1, 29) = 0.17, *p* = 0.69) or interaction (Sex × Condition, *F*(1, 29) = 1.62, *p* = 0.21) on the time spent close to the primary imprinting stimulus. The chicks significantly remained close the primary imprinting stimulus (*t*(32) = 87.18, *p* < 0.001, Cohen’s *d* = 15.18) 97% of their time (± 0.54 SEM).

All chicks (33) remained significantly more on the side of the arena, where the primary imprinting stimulus was displayed (Table S3 in the Supplementary Material).

#### Secondary imprinting

There were non-significant effects of Condition on the time spent close to the secondary imprinting stimulus (*F*(1, 29) = 0.14, *p* = 0.72), Sex (*F*(1, 29) = 0.49, *p* = 0.49) or interaction (Sex × Condition, *F*(1, 29) = 0.70, *p* = 0.41) on the time spent close to the secondary imprinting stimulus. The chicks significantly remained close the secondary imprinting stimulus (*t*(32) = 34.72, *p* < 0.001, Cohen’s *d* = 6.04) 93% of their time (± 1.25 SEM).

All the chicks (33) remained significantly more on the side of the arena, where the secondary imprinting stimulus was displayed (Table S3 in the Supplementary Material).

#### Testing

**Figure 3:**
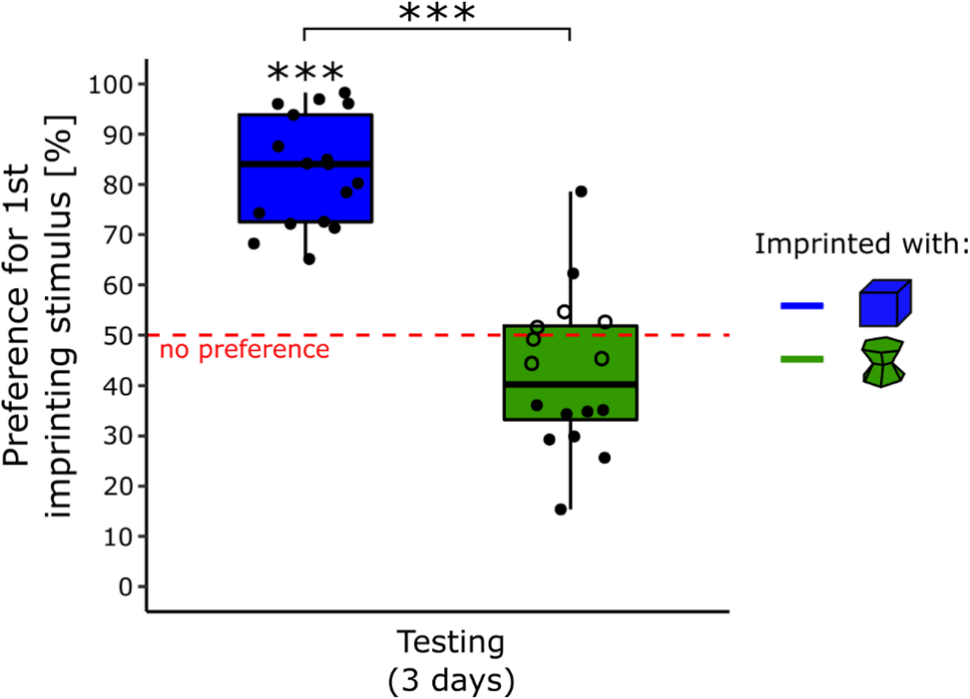
In experiment 3, the object used during the primary imprinting phase strongly influenced chicks’ preference for it when exposed to a novel stimulus (p < 0.001, ***). The blue boxplot represents the preference score of the chicks imprinted with the blue stimulus. The green boxplot represents the preference score of the chicks imprinted with the green stimulus. Filled dots show the individuals having a significant preference while empty dots show the individuals having no preference. The asterisks represent the overall significance of each condition, against no preference and between conditions.

The results are shown in Fig. 3. There was a significant effect of Condition (*F*(1, 29) = 70.35, *p* < 0.001) but non-significant effects of Sex (*F*(1, 28) = 2.98, *p* = 0.095), Day (*F*(2, 58) = 0.54, *p* = 0.59) or interactions (Sex × Condition, *F*(1, 29) = 1.21, *p* = 0.28; Sex × Day, *F*(2, 58) = 0.072, *p* = 0.93, Condition × Day, *F*(2, 58) = 0.41, *p* = 0.67; Sex × Condition × Day, *F*(2, 58) = 0.010, *p* = 0.10) on the preference for the primary imprinting stimulus.

The preference for the primary imprinting stimulus was significantly different from chance level for the chicks imprinted with the blue stimulus (*t*(16) = 12.27, *p* < 0.001, Cohen’s *d* = 2.98, *Bonferroni* correction) with an average time spent close to the primary imprinting stimulus of 83% (± 2.66 SEM). The Preference score was non-significantly different from chance level for the chicks imprinted with the green stimulus (*t*(15) =  − 1.94, *p* = 0.14, Cohen’s *d* = 0.48, Bonferroni correction) with an average time spent close to the primary imprinting stimulus of 42% (± 3.90 SEM).

All the chicks (17) had a significant preference for the imprinting stimulus while primary imprinted with the blue stimulus (Table S3 in the Supplementary Material). Whereas for the chicks primarily imprinted with the green stimulus, 2 (13%) had a significant preference for their primary imprinting stimulus, 6 (37%) had no preference and 8 (50%) had a preference for the unfamiliar stimulus (Table S3 in the Supplementary Material). Levene’s test showed that the variances of the two conditions were similar (*F*(1, 31) = 1.45, *p* = 0.24).

### Experiment 4

#### Primary imprinting

There were non-significant effects Condition (*F*(1, 29) = 3.44, *p* = 0.074), Sex, (*F*(1, 29) = 0.50, *p* = 0.23) or interaction (Sex × Condition, *F*(1, 29) = 0.10, *p* = 0.75) on the time spent close to the primary imprinting stimulus. The chicks significantly remained close the primary imprinting stimulus (*t*(32) = 45.53, *p* < 0.001, Cohen’s *d* = 7.93) 95% of their time (± 0.99 SEM).

Individual preferences were calculated and showed that 32 (97%) chicks remained significantly more on the side of the arena, where the primary imprinting stimulus was displayed, and 1 (3%) did not (Table S4 in the Supplementary Material).

#### Secondary imprinting

There were non-significant effects of Condition on the time spent close to the secondary imprinting stimulus (*F*(1, 29) = 0.27, *p* = 0.61), Sex (*F*(1, 29) = 0.002, *p* = 0.96) or interaction (Sex × Condition, *F*(1, 29) = 0.30, *p* = 0.59) on the time spent close to the secondary imprinting stimulus. The chicks significantly remained close the secondary imprinting stimulus (*t*(32) = 40.27, *p* < 0.001, Cohen’s *d* = 7.01) 93% of their time (± 1.07 SEM).

All chicks (33) chose significantly more the side of the arena where the secondary imprinting stimulus was displayed (Table S4 in the Supplementary Material).

#### Testing

**Figure 4:**
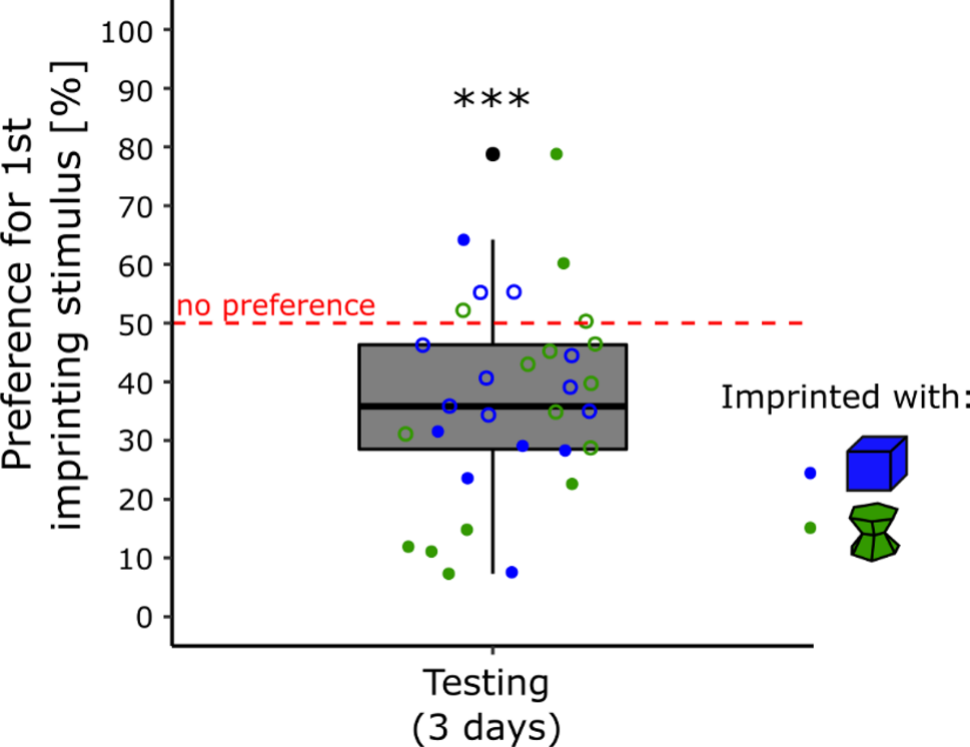
In experiment 4, chicks of both conditions had a preference for the secondary imprinting objects (p < 0.001, ***). The blue dots represent the preference score of the chicks imprinted with the blue stimulus. The green dots represent the preference score of the chicks imprinted with the green stimulus. Filled dots show the individuals having a significant preference while empty dots show the individuals having no preference. The asterisks represent the overall significance of both conditions pooled, against no preference.

Two chicks (2 males of the blue condition) were removed from the following analyses because the video recordings of their last testing day went missing (camera crash). The results are shown in Fig. 4. There were non-significant effects of Condition (*F*(1, 27) = 0.11, *p* = 74), Sex (*F*(1, 27) = 2.22, *p* = 0.15), Day (*F*(2, 54) = 0.14, *p* = 0.87) or interactions (Sex × Condition, *F*(1, 27) = 0.16, *p* = 0.69; Sex × Day, *F*(2, 54) = 0.21, *p* = 0.81, Condition × Day, *F*(2, 54) = 0.38, *p* = 0.68; Sex × Condition × Day, *F*(2, 54) = 0.50, *p* = 0.61) on the preference for the primary imprinting stimulus. The preference for the primary imprinting stimulus was significantly different from chance-level (*t*(30) =  − 4.24, *p* < 0.001, Cohen’s *d* = 0.76) with an average time spent close to the secondary imprinting stimulus of 63% (± 3.05 SEM).

In the blue condition, 1 chick (7%) had a significant preference for the primary imprinting stimulus, 9 (60%) had no preference, and 5 (33%) significantly preferred the unfamiliar stimulus. In the green condition, 2 chicks (13%) had a significant preference for the primary imprinting stimulus, 9 (56%) had no preference, and 5 (31%) had a significant preference for the unfamiliar stimulus (Table S4 in the Supplementary Material). Levene’s test showed that the variances of the two conditions were similar (*F*(1, 29) = 2.15, *p* = 0.15).

## Discussion

Due to the difficulties in assessing animals behaviours over prolonged durations, the temporal stability and individual variability of social attachment in filial imprinting have remained unexplored. To understand more about it, we used an automated behavioural tracking method and followed the animals’ preferences for familiar and novel stimuli for 6 consecutive days. The temporal stability of the imprinting preferences was investigated by manipulating the duration of the imprinting and the stimuli used. When imprinted for 14 h over 1 day (Experiment 1), the chicks exhibited an unsteady preference for their imprinting stimulus compared to when exposed for 42 h over 3 days (Experiment 2). In fact, after 1 day of imprinting, the filial preferences were disparate between conditions. While the chicks of the blue condition always had a preference for their imprinting stimulus at testing, the chicks of the green condition lost their significant preference for the imprinting stimulus on the fourth testing day. They started to explore more the unfamiliar stimulus (blue stimulus). Since we know that chicks mainly rely on colour to recognise their artificial imprinting objects^38^, this difference confirms previous reports of an advantage of blue over green imprinting stimuli^40–42^. In contrast, after 3 days of imprinting, chicks of both conditions had a robust and stable preference for their imprinting objects. Moreover, we excluded the possibility that the difference observed was affected by the time spent close to the imprinting stimuli by showing that bot conditions spent the same amount of time close to their respective stimulus during the imprinting phase.

The preference observed in Experiment 1 for the imprinting stimulus across days in the blue condition and on the first testing day of the green condition indicated that chicks imprinted on their respective stimuli. Nevertheless, 14 h of imprinting are insufficient to produce a robust and stable imprinting preference for artificial stimuli. The unlearned preferences are influencing the animals’ filial preferences. Therefore, the decrease of preference for the imprinting stimulus in the green condition suggests that the blue stimulus is more attractive to the chicks. Due to repeated testing, secondary imprinting with the blue stimulus might take place in the green condition. This would explain why the animals spend more time close to it rather than a general lack of memory (the preference is stabilised at chance-level in the green condition and blue-imprinted chicks steadily remembered and prefered the imprinting stimulus). The difference between blue and green-imprinted chicks is apparent also looking at the individual performances. More than half of the chicks had a preference for the imprinting stimulus, and only 6% had a preference for the novel stimulus in the blue-imprinted chicks. In contrast, only less than half of the chicks had a preference for the imprinting stimulus, and 19% preferred the novel stimulus in the green-imprinted chicks.

Several biochemicals changes associated with imprinting have been described later than 15 h after the start of the imprinting process, confirming the idea that imprinting might not be fully consolidated on the first day of exposure^12^. Furthermore, the mechanisms responsible for the spontaneous preferences observed in chicks strongly influence the imprinting memory^25,26^. In Experiment 1, it seems that after 14 h of exposure to a stimulus, the imprinting memories are available but not fully consolidated yet. The preferences also seem more plastic after imprinting with less predisposed stimuli.

Hence, because the same experience produces different learning outcomes, it appears that predispositions affect both learning and the between-subjects variability in learning, with faster and stronger learning and less variability when subjects are exposed to predisposed stimuli.

The analysis of individual behaviours revealed that some chicks had consistent preferences for unfamiliar stimuli not only at the very beginning of imprinting, as hypothesised by Bateson’s model^46^. By increasing the chicks’ exposure to their imprinting objects to 42 h over 3 days, we observed more robust and stable filial preferences with time for both stimuli (Experiment 2) but still a higher inter-individual variability within the green-imprinted chicks. These results are in line with previous experiments in which preferences for unfamiliar objects have been observed even after 3 days of imprinting in males^16,17^. As stated in the introduction, males are more inclined to approach unfamiliar conspecifics than females, usually showing a strong attachment to their conspecifics^53,54^. In this study, the filial preference was similar in both sexes.

More prolonged imprinting exposure has been associated with stronger preference scores for the imprinting stimulus^4,20^. Furthermore, our study suggests that the imprinting duration strongly influences the filial preference steadiness. After 42 h over 3 days of exposure to an object, the imprinting memory appears to be consolidated for both artificial stimuli (green and blue). Nonetheless, animals’ spontaneous preferences for specific stimuli are still, to a lower degree, influencing chicks’ filial preferences. The variability within the green condition (less predisposed colour) was three-time higher than in the blue condition. While almost all chicks showed a strong preference for their imprinting objects in the blue condition, more than a third did not prefer their imprinting stimulus in the green condition.

The evidence that prolonged exposure to an object leads to more stable preferences in time is convincing and in line with previous evidence^20,43^. Nevertheless, the ontogenetic stage at which the preferences were tested could have influenced filial preferences. In the third experiment, we assessed whether this was the case. As in the first experiment, both conditions (blue and green) were exposed to their respective objects for 14 h (day 1), but this time, their filial preference was tested from day 4 to day 6, after exposure to a novel object on day 2 and 3 (this prevented a complete ‘social’ deprivation). Similarly to what observed in the first experiment (short imprinting duration), the filial preferences observed differed between conditions. In the blue condition, all the individuals preferred their imprinting object, showing that the memory of the imprinting stimulus lasted although chicks had been detached by the initial stimulus for days. At the same time, preferences among individuals of the green conditions were disparate with 13% of the individuals preferring the imprinting object, 37% showing no preferences and even 50% showing a preference for the novel object. Interestingly, the preferences observed here were not wholly similar to the first experiment. The preferences observed in both conditions were stable in time. Then, one could argue that the filial preferences observed resulted from a lack of memory, but the different patterns of preference between conditions and the literature suggest otherwise. In the case of a memory loss, chicks would have either approached the more attractive stimulus (blue object) or not chosen any. However, the results showed both patterns depending on the primary imprinting stimulus used. Moreover, studies exploring successive imprinting always described a recall of the primary imprinting object^21,62^.

In Experiment 4, we assessed whether chicks had a preference for their primary imprinting stimulus compared to their secondary imprinting stimulus during the testing phase. Both conditions showed a similar preference for the secondary imprinting stimulus. As previously shown, chicks can imprint on multiple objects^22,23^. Furthermore, a preference for a primary imprinting stimulus can be reversed after prolonged exposure with a secondary imprinting object^63^, which is in line with the experimental settings used here (1 day of primary imprinting and 2 days of secondary imprinting). It is then very likely that the filial bond formed with the secondary imprinting object has influenced the chicks’ filial preferences toward their primary imprinting stimulus.

In all experiments, the filial imprinting preferences were all pointing in the same direction. Overall, chicks of the blue condition (where blue is a more predisposed colour) had a more robust and stable preference in time for their imprinting stimulus than the chicks of the green condition (where green is a less predisposed colour). The differences between conditions were not the result of the time spent close to their respective objects during imprinting, given that chicks engaged with the imprinting stimuli for the same amount of time. This strongly suggests that some features of the objects (e.g. colour) are more efficient for forming filial imprinting preferences. Further studies should be performed to understand the influence of colour in comparison to shape.

Altogether, our results indicate that the temporal stability of filial imprinting preferences is influenced by the amount of experience (exposure duration and successive imprinting) and spontaneous preferences (predispositions). Moreover, using automated tracking for assessing chicks’ behaviour for several days, we show that chicks with similar experiences can have steady and robust idiosyncratic differences in their preferences for familiar vs novel stimuli. Some chicks consistently preferred to approach their imprinting stimulus, while others preferred the unfamiliar stimulus, even if they had the same experience. Moreover, this consistent inter-individual variability (a phenomenon already documented in other animal species, such as fruit flies)^59,64–68^ was modulated by the animals’ spontaneous preferences. Further studies should clarify whether these differences stem from genetic variability and/or derive from stochasticity in the course of development^69^, as well as their neurobiological basis.

## Materials and methods

### Ethical note

This study was carried out in compliance with the European Union and the Italian law on the treatment of animals. The experimental procedures were performed in accordance with the ARRIVE guidelines and approved by the Ethical Committee of the University of Trento and licenced by the Italian Health Ministry (permit number 53/2020).

### Subjects

We used 128 domestic chicks (*Gallus gallus*) of the strain Ross 308 (a strain selected to be sexually dimorphic at birth, based on the feathers). The eggs were coming from a commercial hatchery (Azienda Agricola Crescenti) and were incubated at the University of Trento under standards controlled conditions (37.7 °C and 40% of humidity). Three days before hatching eggs were transferred into opaque individual boxes within a hatching chamber (37.7 °C and 60% of humidity).

### Setup

**Figure 5:**
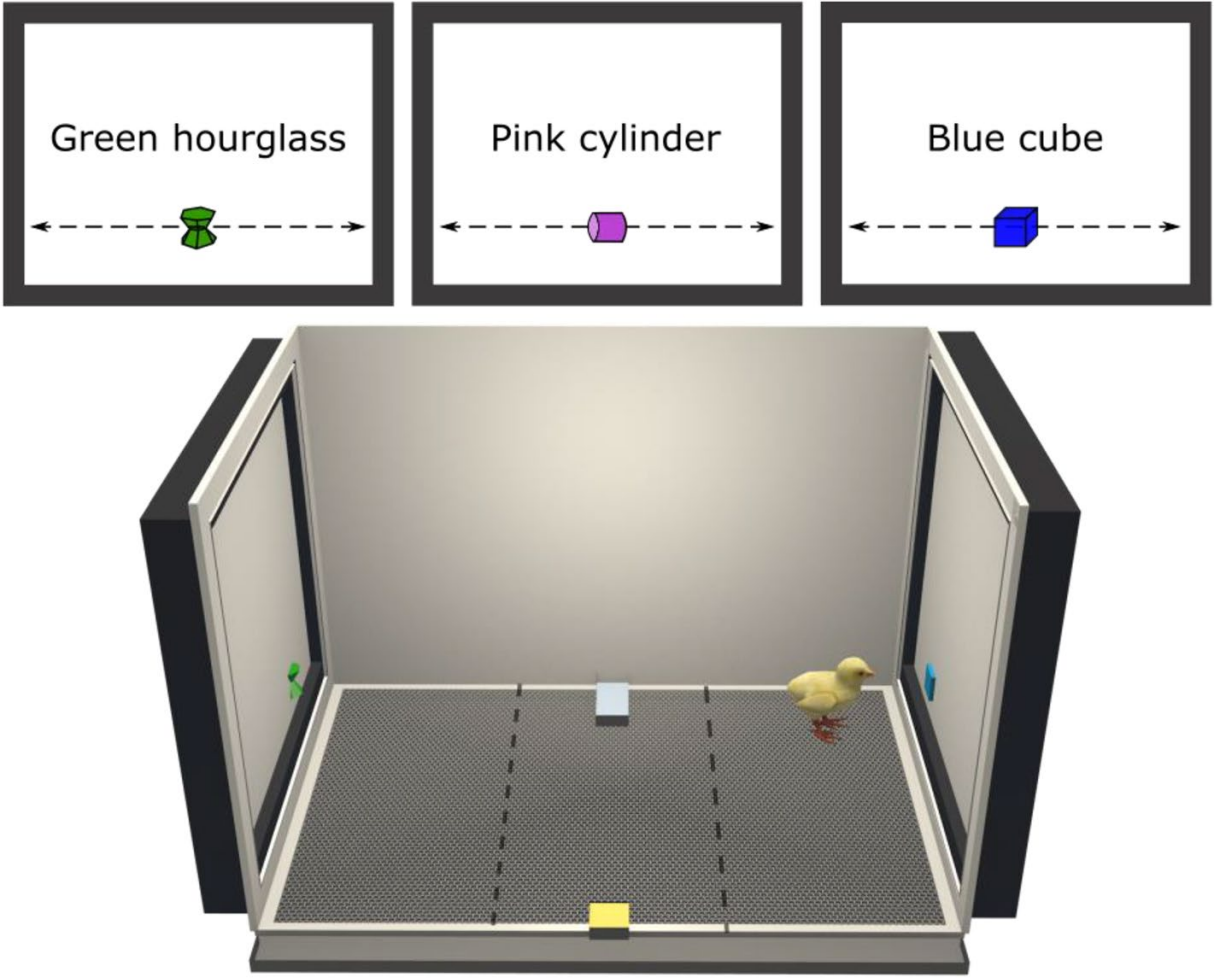
Three-dimensional representation of the apparatus and stimuli used in this study (created with Blender 2.8). The stimuli were moving horizontally alongside the screens to attract the attention of the animals. The filial preference of a chick was revealed by its choice to remain near the stimuli displayed. The dashed lines show the delimitation of the virtual zones used to assess the preference of the animal. The time spent near the stimuli was monitored to calculate a Preference for the imprinting stimuli.

Several apparatuses were used simultaneously. Each apparatus had a rectangular shape (90 cm × 60 cm × 60 cm, Fig. 5). A high-frequency computer screen (ASUS MG248QR, 120 Hz) was located on each smaller wall and used to display stimuli. A Microsoft life camera was located on the top of the apparatus at 105 cm from the ground to record the behaviours of the animal. Food and water were located in the middle of the apparatus and available ad libitum.

### Stimuli

Three-dimensional virtual visual stimuli were created (Fig. 5) and animated on Blender (v2.79). The objects were different in term of colours and shapes (green hourglass, hex: 30B619; blue cube, hex: 2EBAFF; pink cylinder, hex: C33CDB) but had similar sizes (5 cm × 5 cm, Fig. 5). The stimuli were animated (linear movement) in a 3D environment and were crossing the screen in 4.5 s (from left to right). The video displaying the stimuli was exported with a high frame frequency (120 frames per second, fps).

### General procedure

After hatching, chicks were sexed in darkness and were transported in another room and individually placed in their apparatus for 6 days in a day-night cycle (14:10 h). During the day, the chicks were exposed to the stimuli displayed on the screens. The displaying of the stimuli was divided into different sessions depending on the experimental phase (from 2 h to 30 min). The position of the stimuli on the screens was counterbalanced across sessions. During the night, dark screens were displayed. Four different experiments were performed. Each experiment was divided into 2 or 3 distinct phases (primary imprinting, secondary imprinting and testing) and conditions (blue and green). The duration of each phase was manipulated from one experiment to another. Chicks were donated to local farms at the end of the experiment.

#### Primary imprinting

This phase was the first one of each experiment. The chicks were exposed to a single imprinting stimulus (the blue or the green depending on the condition). The imprinting sessions lasted 2 h (7 sessions) on the first primary imprinting day and 1 h on the following days (13 sessions interrupted by 5 min period of dark screens).

#### Secondary imprinting

In Experiments 3 and 4, this phase followed the primary imprinting phase and lasted 2 days. The chicks were exposed to a new stimulus (a pink cylinder). The sessions were lasting 1 h (13 sessions interrupted by 5 min period of dark screens).

#### Testing

Depending on the experiment, the testing phase was either following the primary (Experiment 1 and 2) or secondary imprinting phase (Experiment 3 and 4). The chicks were exposed to two stimuli (primary imprinting stimulus vs novel stimulus or primary vs secondary imprinting stimulus), and their preferences were monitored. The sessions lasted thirty minutes (24 sessions interrupted by 5 min period of dark screens between each session).

### Experiment 1

**Figure 6:**
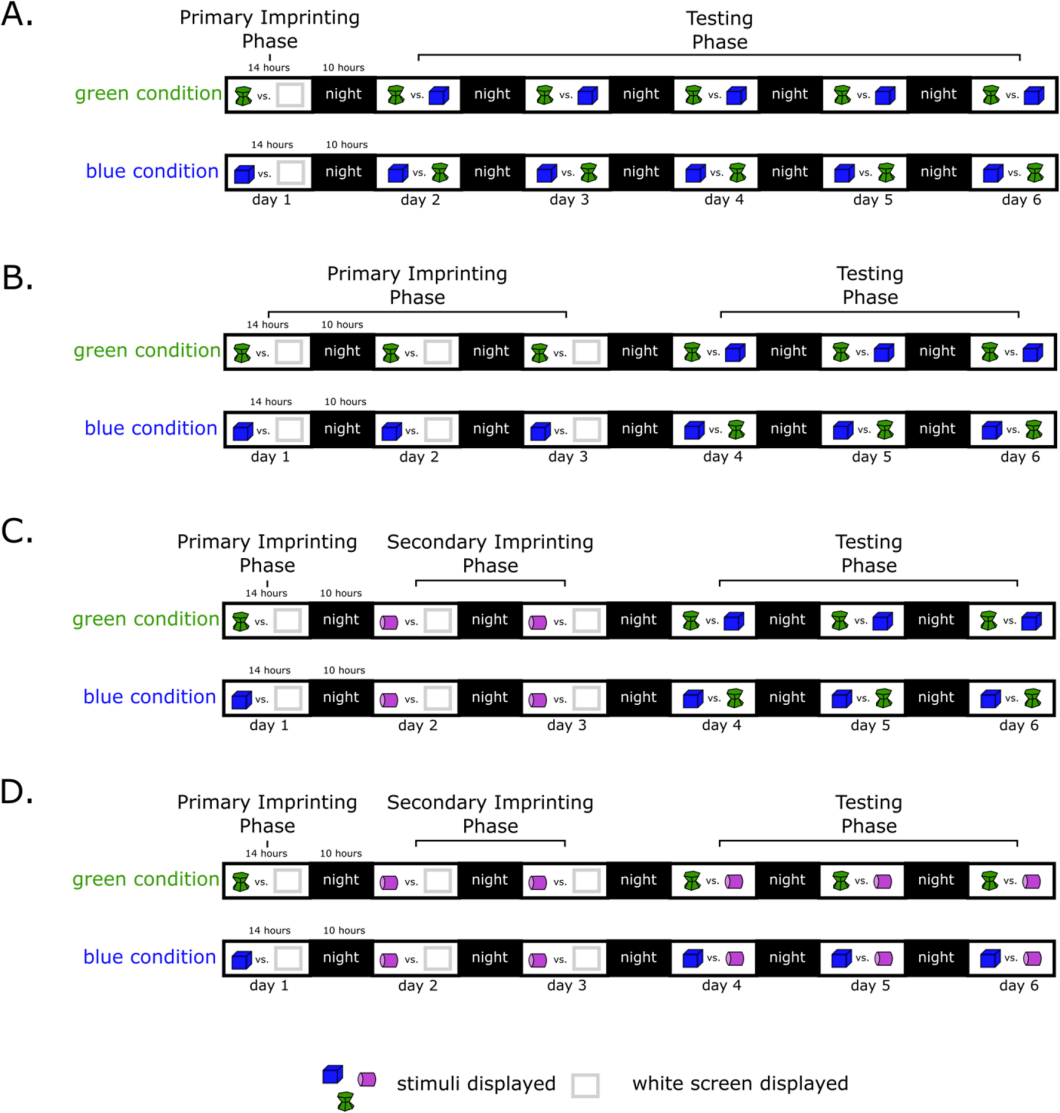
Experimental timelines of experiment 1 (**A**), 2 (**B**), 3 (**C**) and 4 (**D**).

Chicks were exposed to an imprinting stimulus for 1 day (blue or green stimulus depending on the condition) and then tested with two stimuli (imprinting stimulus vs unfamiliar stimulus) for 5 days (Fig. 6A).

#### Subjects

We imprinted 16 animals (8 females, 8 males) with the green hourglass (green condition) and 16 animals (8 females, 8 males) with the blue cube (blue condition).

### Experiment 2

Chicks were exposed to an imprinting stimulus for 3 days (blue or green stimulus depending on the condition) and then tested with two stimuli (imprinting stimulus vs unfamiliar stimulus) for 3 days (Fig. 6B).

#### Subjects

We imprinted 16 animals (8 females, 8 males) with the green hourglass (green condition) and 16 animals (8 females, 8 males) with the blue cube (blue condition).

### Experiment 3

Chicks were exposed to a primary imprinting stimulus for 1 day (blue or green stimulus depending on the condition), secondary imprinting stimulus (pink stimulus) for 2 days and then tested with two stimuli (primary imprinting stimulus vs unfamiliar stimulus) for 3 days (Fig. 6C).

#### Subjects

We imprinted 16 animals (8 females, 8 males) with the green hourglass (green condition) and 16 animals (8 females, 8 males) with the blue cube (blue condition).

### Experiment 4

Chicks were exposed to a primary imprinting stimulus for 1 day (blue or green stimulus depending on the condition), secondary imprinting stimulus (pink stimulus) for 2 days and then tested with two stimuli (primary imprinting stimulus vs secondary imprinting stimulus) for 3 days (Fig. 6D).

#### Subjects

We imprinted 16 animals (8 females, 8 males) with the green hourglass (green condition) and 17 animals (8 females, 9 males) with the blue cube (blue condition).

### Data analysis

The position of the animal was analysed automatically using DeepLabCut, an open-source deep-learning toolbox made to track efficiently animal behaviours^61^. The preference for a stimulus was assessed using the time spent inside the closest zone to it (30 cm wide). The apparatus had been virtually divided into three equal zones corresponding to the left, centre and right side of each arena (Fig. 5).

#### Imprinting phases

During these phases (primary and secondary), the number of seconds [s] spent close to the stimulus (in the 30 cm zone close to the screen) was analysed to check for the amount of time spent attending the imprinting object.

#### Testing phase

For this phase, the Preference for the imprinting stimulus [%] was calculated using the following formula:

$$ {\text{Preference for the imprinting stimulus}} = \frac{{\text{Time spent close primary imprinting stimulus}}}{{\text{Time spent close both screens}}} \times 100. $$

Using this formula, a score of 50% indicates no preference for either stimulus. A score higher than 50% indicates more time spent at the primary imprinting object. A score lower than 50% indicates more time spent at the unfamiliar stimulus (Experiment 1, 2 and 3) or the secondary imprinting object (Experiment 4).

### Statistical analysis

#### Imprinting phases

To assess the time spent by the chicks close to the imprinting stimulus during the imprinting phases (primary and secondary), we used an ANOVA with seconds spent close to the imprinting stimulus as dependent variable and Condition (imprinted with green, imprinted with blue), Sex (female, male). In all experiments, data met assumptions of parametric analyses.

#### Testing phase

To determine whether chicks had different preferences for the imprinting stimulus (or the primary imprinting stimulus) between Condition (imprinted with green, imprinted with blue), Sex (female, male) and Day (experiment 1: day 2, 3, 4, 5, 6; other experiments: day 4, 5, 6), we performed a mixed-design ANOVA for each testing phase. To meet parametric analysis assumptions (visualised using Q-Q plots), we arcsin transformed the data. To check whether chicks had a significant preference for the imprinting stimulus or unfamiliar stimulus (primary vs secondary imprinting stimulus in experiment 4) we performed two-tailed one-sample t-tests vs the chance level (50%). Since the chicks underwent several imprinting and testing sessions across testing days, it was possible to test their preference individually. Individual preferences were assessed and compared from chance-level (50%) using two-tailed one-sample t-tests. In each experiment, Levene’s test was conducted to explore chicks variability between conditions (imprinted with green or imprinted with blue). For all experiments, we used an α = 0.05. Analyses were performed using RStudio v1.1^70^. The following packages were used: *goftest*^71^, *nlme*^72^, *lme*^73^, *tidyr*^74^, *plyr*^75^, *dplyr*^76^, *reshape*^77^, *lsr*^78^*, ggplot2*^79^.

## Supplementary Information

Supplementary Information.

## Data Availability

The datasets (.csv) are available on Fig Share (https://doi.org/10.6084/m9.figshare.12074565).

## References

[1] Versace E, Vallortigara G (2015). Origins of knowledge: Insights from precocial species. Front. Behav. Neurosci..

[2] Bateson PPG (1966). The characteristics and context of imprinting. Biol. Rev..

[3] Bolhuis JJ (1991). Mechanisms of avian imprinting: A review. Biol. Rev..

[4] Hess EH (1959). Imprinting. Science.

[5] Lorenz KZ (1937). The companion in the bird’s world. Auk.

[6] McCabe BJ (2019). Visual imprinting in birds: Behavior, models, and neural mechanisms. Front. Physiol..

[7] Spalding DA (1873). Instinct, with Original Observations of Young Animals.

[8] Vallortigara G, Versace E, Vonk J, Shackelford T (2018). Filial imprinting. Encyclopedia of Animal Cognition and Behavior.

[9] Di Giorgio E (2017). Filial responses as predisposed and learned preferences: Early attachment in chicks and babies. Behav. Brain Res..

[10] Rose SPR (2000). God’s organism? The chick as a model system for memory studies. Learn. Mem..

[11] Rose SPR (2003). The Making of Memory: From Molecule to Mind.

[12] Solomonia RO, McCabe BJ (2015). Molecular mechanisms of memory in imprinting. Neurosci. Biobehav. Rev..

[13] Nicol CJ (2015). The Behavioural Biology of Chickens.

[14] Chiandetti C, Vallortigara G (2011). Intuitive physical reasoning about occluded objects by inexperienced chicks. Proc. R. Soc. B Biol. Sci..

[15] Versace E, Schill J, Nencini AMM, Vallortigara G (2016). Naïve chicks prefer hollow objects. PLoS ONE.

[16] Versace E, Regolin L, Vallortigara G, Versace E, Regolin L, Vallortigara G (2006). Emergence of grammar as revealed by visual imprinting in newly-hatched chicks. The Evolution of Language.

[17] Versace E, Spierings MJ, Caffini M, Ten Cate C, Vallortigara G (2017). Spontaneous generalization of abstract multimodal patterns in young domestic chicks. Anim. Cogn..

[18] Santolin C, Rosa-Salva O, Vallortigara G, Regolin L (2016). Unsupervised statistical learning in newly hatched chicks. Curr. Biol..

[19] Wood SMW, Wood JN (2015). A chicken model for studying the emergence of invariant object recognition. Front. Neural Circuits..

[20] Bateson PPG, Jaeckel JB (1976). Chicks’ preferences for familiar and novel conspicuous objects after different periods of exposure. Anim. Behav..

[21] Salzen EA, Meyer CC (1968). Reversibility of imprinting. J. Comp. Physiol. Psychol..

[22] Boakes R, Panter D (1985). Secondary imprinting in the domestic chick blocked by previous exposure to a live hen. Anim. Behav..

[23] Bolhuis JJ, Trooster WJ (1988). Reversibility revisited: Stimulus-dependent stability of filial preference in the chick. Anim. Behav..

[24] Johnson MH, Bolhuis JJ, Horn G (1985). Interaction between acquired preferences and developing predispositions during imprinting. Anim. Behav..

[25] Miura M, Matsushima T (2016). Biological motion facilitates filial imprinting. Anim. Behav..

[26] Miura M, Nishi D, Matsushima T (2020). Combined predisposed preferences for colour and biological motion make robust development of social attachment through imprinting. Anim. Cogn..

[27] Lemaire BS (2020). No evidence of spontaneous preference for slowly moving objects in visually naïve chicks. Sci. Rep..

[28] Rosa-Salva O, Hernik M, Broseghini A, Vallortigara G (2018). Visually-naïve chicks prefer agents that move as if constrained by a bilateral body-plan. Cognition.

[29] Vallortigara G (2012). Core knowledge of object, number, and geometry: A comparative and neural approach. Cogn. Neuropsychol..

[30] Johnson MH, Horn G (1988). Development of filial preferences in dark-reared chicks. Anim. Behav..

[31] Rosa-Salva O, Mayer U, Vallortigara G (2019). Unlearned visual preferences for the head region in domestic chicks. PLoS ONE.

[32] Rosa-Salva O, Regolin L, Vallortigara G (2010). Faces are special for newly hatched chicks: Evidence for inborn domain-specific mechanisms underlying spontaneous preferences for face-like stimuli. Dev. Sci..

[33] Miura M, Matsushima T (2012). Preference for biological motion in domestic chicks: Sex-dependent effect of early visual experience. Anim. Cogn..

[34] Vallortigara G, Regolin L, Marconato F (2005). Visually inexperienced chicks exhibit spontaneous preference for biological motion patterns. PLoS Biol..

[35] Rosa-Salva O, Grassi M, Lorenzi E, Regolin L, Vallortigara G (2016). Spontaneous preference for visual cues of animacy in naïve domestic chicks: The case of speed changes. Cognition.

[36] Versace E, Ragusa M, Vallortigara G (2019). A transient time window for early predispositions in newborn chicks. Sci. Rep..

[37] Guhl AM, Ortman LL (1953). Visual patterns in the recognition of individuals among chickens. Condor.

[38] Maekawa F (2006). Imprinting modulates processing of visual information in the visual wulst of chicks. BMC Neurosci..

[39] Ham AD, Osorio D (2007). Colour preferences and colour vision in poultry chicks. Proc. R. Soc. B Biol. Sci..

[40] Kovach JK (1971). Effectiveness of different colors in the elicitation and development of approach behavior in chicks. Behaviour.

[41] Salzen EA, Lily RE, McKeown JR (1971). Colour preference and imprinting in domestic chicks. Anim. Behav..

[42] Schaefer HH, Hess EH (2010). Color preferences in imprinting objects. Z. Tierpsychol..

[43] Bolhuis JJ, Cook S, Horn G (2000). Getting better all the time: Improving preference scores reflect increases in the strength of filial imprinting. Anim. Behav..

[44] Jackson S, Bateson PPG (1974). Imprinting and exploration of slight novelty in chicks. Nature.

[45] Versace E, Fracasso I, Baldan G, Dalle Zotte A, Vallortigara G (2017). Newborn chicks show inherited variability in early social predispositions for hen-like stimuli. Sci. Rep..

[46] Bateson PPG (1973). Preferences for familiarity and novelty: A model for the simultaneous development of both. J. Theor. Biol..

[47] Bateson PPG (1979). Brief exposure to a novel stimulus during imprinting in chicks and its influence on subsequent preferences. Anim. Learn. Behav..

[48] Chantrey DF (1974). Stimulus preexposure and discrimination learning by domestic chicks: Effect of varying interstimulus time. J. Comp. Physiol. Psychol..

[49] Honey RC, Bateson P (1996). Stimulus comparison and perceptual learning: Further evidence and evaluation from an imprinting procedure. Q. J. Exp. Psychol. Sect. B Comp. Physiol. Psychol..

[50] Jackson C (2008). Dynamics of a memory trace: Effects of sleep on consolidation. Curr. Biol..

[51] Solomonia RO (2003). Analysis of differential gene expression supports a role for amyloid precursor protein and a protein kinase C substrate (MARCKS) in long-term memory. Eur. J. Neurosci..

[52] Solomonia RO (2011). Mitochondrial proteins, learning and memory: Biochemical specialization of a memory system. Neuroscience.

[53] Vallortigara G (1992). Affiliation and aggression as related to gender in domestic chicks (*Gallus gallus*). J. Comp. Psychol..

[54] Vallortigara G, Andrew RJ (1991). Lateralization of response by chicks to change in a model partner. Anim. Behav..

[55] Smith FV, Templeton WB (1966). Genetic aspects of the response of the domestic chick to visual stimuli. Anim. Behav..

[56] Gribovskiy, A. *et al.**Automated Analysis of Behavioural Variability and Filial Imprinting of Chicks (Gallus gallus), Using Autonomous Robots* (2015).

[57] Zidar J, Balogh ACV, Leimar O, Løvlie H (2019). Generalization of learned preferences covaries with behavioral flexibility in red junglefowl chicks. Behav. Ecol..

[58] Anderson DJJ, Perona P (2014). Toward a science of computational ethology. Neuron.

[59] Versace E, Caffini M, Werkhoven Z, de Bivort BL (2020). Individual, but not population asymmetries, are modulated by social environment and genotype in *Drosophila melanogaster*. Sci. Rep..

[60] Goldman JG, Wood JN (2015). An automated controlled-rearing method for studying the origins of movement recognition in newly hatched chicks. Anim. Cogn..

[61] Nath T (2019). Using DeepLabCut for 3D markerless pose estimation across species and behaviors. Nat. Protoc..

[62] Bolhuis JJ, Bateson P (1990). The importance of being first: A primacy effect in filial imprinting. Anim. Behav..

[63] Cherfas JJ, Scott A (1981). Impermanent reversal of fillial imprinting. Anim. Behav..

[64] Buchanan SM, Kain JS, de Bivort BL (2015). Neuronal control of locomotor handedness in Drosophila. Proc. Natl. Acad. Sci..

[65] Honegger KS, Smith MA-YY, Churgin MA, Turner GC, De Bivort BL (2019). Idiosyncratic neural coding and neuromodulation of olfactory individuality in Drosophila. Proc. Natl. Acad. Sci..

[66] Kain JS, Stokes C, De Bivort BL (2012). Phototactic personality in fruit flies and its suppression by serotonin and white. Proc. Natl. Acad. Sci..

[67] Kain JS (2015). Variability in thermal and phototactic preferences in Drosophila may reflect an adaptive bet-hedging strategy. Evolution (N. Y.).

[68] Linneweber GA (2020). A neurodevelopmental origin of behavioral individuality in the Drosophila visual system. Science.

[69] Mitchell KJ (2018). Innate: How the Wiring of Our Brains Shapes Who We are.

[70] RStudio Team. *RStudio: Integrated Development for R* (2015).

[71] Faraway, J., Marsaglia, G., Marsaglia, J. & Baddeley, A. *goftest: Classical Goodness-of-Fit Tests for Univariate Distributions* (2019).

[72] Pinheiro, J., Bates, D., DebRoy, S., Sarkar, D. & R Core, T. *Linear and Nonlinear Mixed Effects Models* (2020).

[73] Bates D, Mächler M, Bolker B, Walker S (2015). Fitting linear mixed-effects models using lme4. J. Stat. Softw..

[74] Wickham, H. & Lionel, H. *tidyr: Tidy Messy Data* (2020).

[75] Wickham H (2011). The split-apply-combine strategy for data analysis. J. Stat. Softw..

[76] Wickham, H., François, R., Henry, L. & Kirill, M. *dplyr: A Grammar of Data Manipulation* (2020).

[77] Wickham H (2007). Reshaping data with the reshape package. J. Stat. Softw..

[78] Navarro D (2015). Learning Statistics with R: A Tutorial for Psychology Students and Other Beginners.

[79] Wickham H (2016). ggplot2: Elegant Graphics for Data Analysis.

