# Supplementary material for: Stability and individual variability of social attachment in imprinting
Source: Sci Rep. 2021 Apr 12;11:7914. doi: 10.1038/s41598-021-86989-3 (PMC8041793; doi:10.1038/s41598-021-86989-3)

**Stability and individual variability of social attachment in imprinting**

Bastien S. Lemaire^1*^, Daniele Rucco^1^, Mathilde Josserand^1,2^, Giorgio Vallortigara^1^, Elisabetta Versace^3,1,4*^

^1^ Center for Mind and Brain Sciences, University of Trento, Italy

^2^ Ecole Normale Supérieure Lyon, France

^3^ School of Biological and Chemical Sciences, Queen Mary University of London, United Kingdom

^4^Alan Turing Institute, United Kingdom

Authors for correspondence:* [bastien.lemaire@unitn.it](mailto:bastien.lemaire@unitn.it); [e.versace@qmul.ac.uk](mailto:e.versace@qmul.ac.uk)


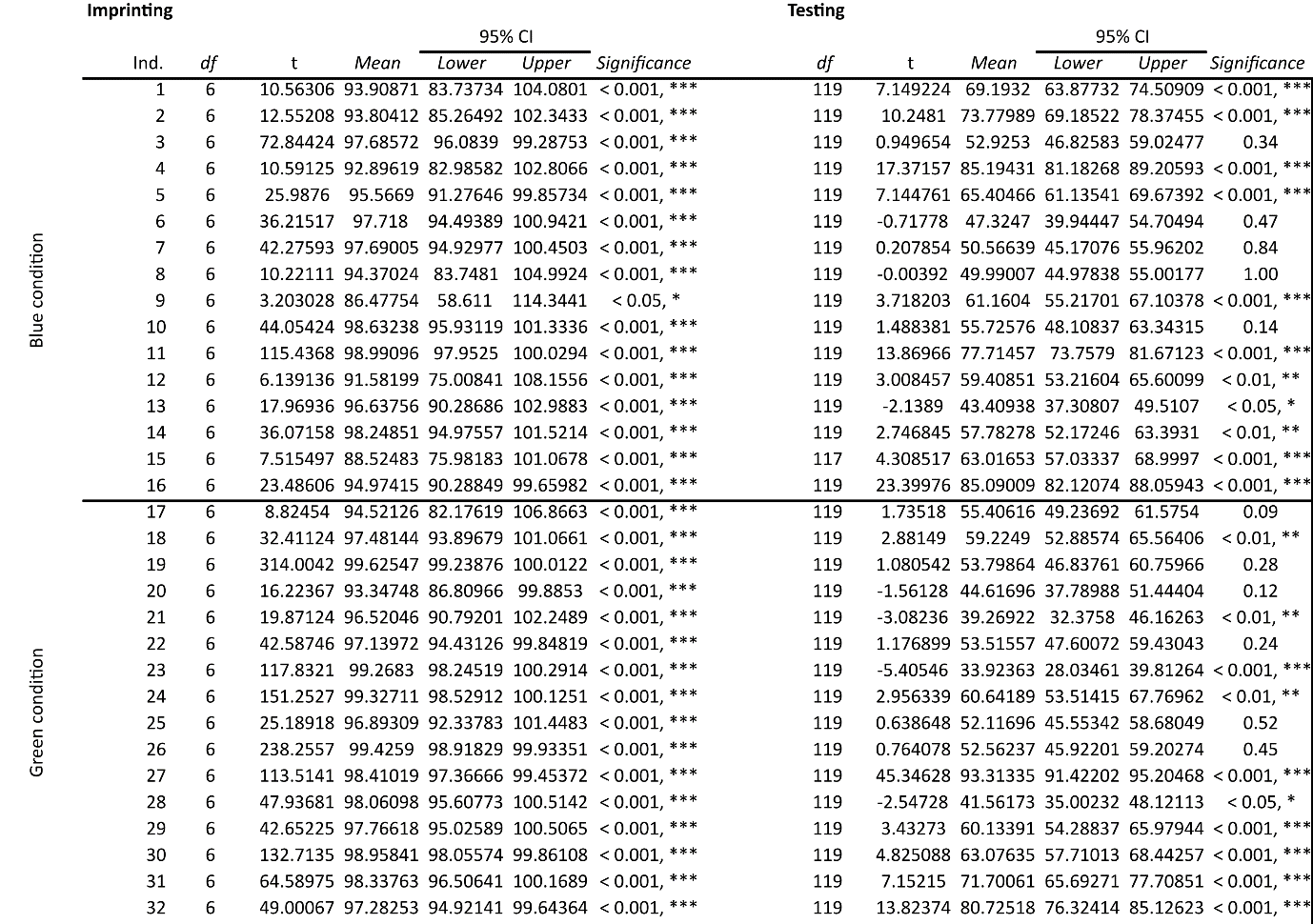
Table 1: Table showing the results of the t-tests on the preference for the imprinting stimulus against chance-level (50%) for each individual of the first experiment.


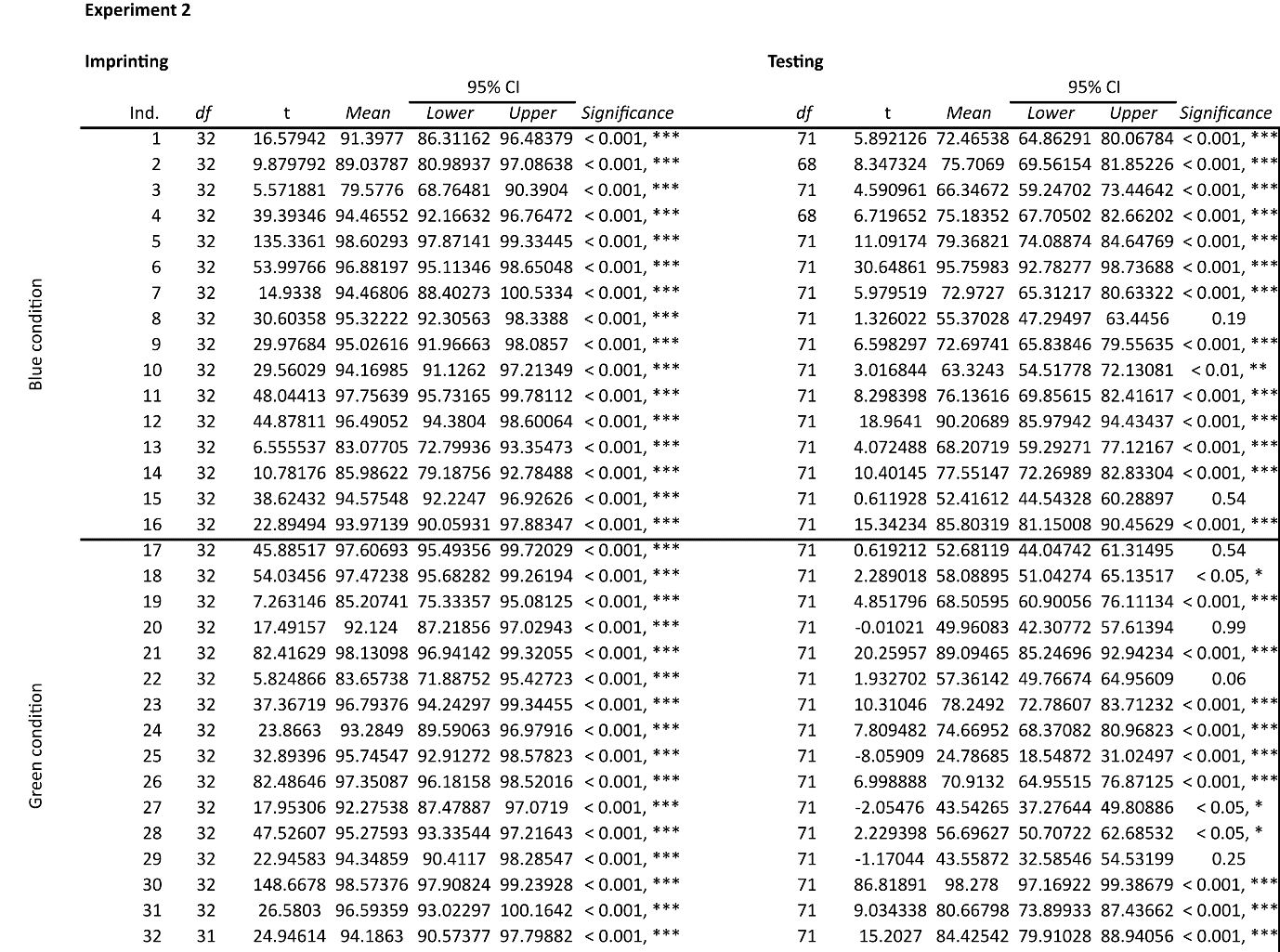
Table 2: Table showing the results of the t-tests on the preference for the imprinting stimulus against chance-level (50%) for each individual of the second experiment.

Table 3: Table showing the results of the t-tests on the preference for the primary imprinting stimulus against chance-level (50%) for each individual of the third experiment.


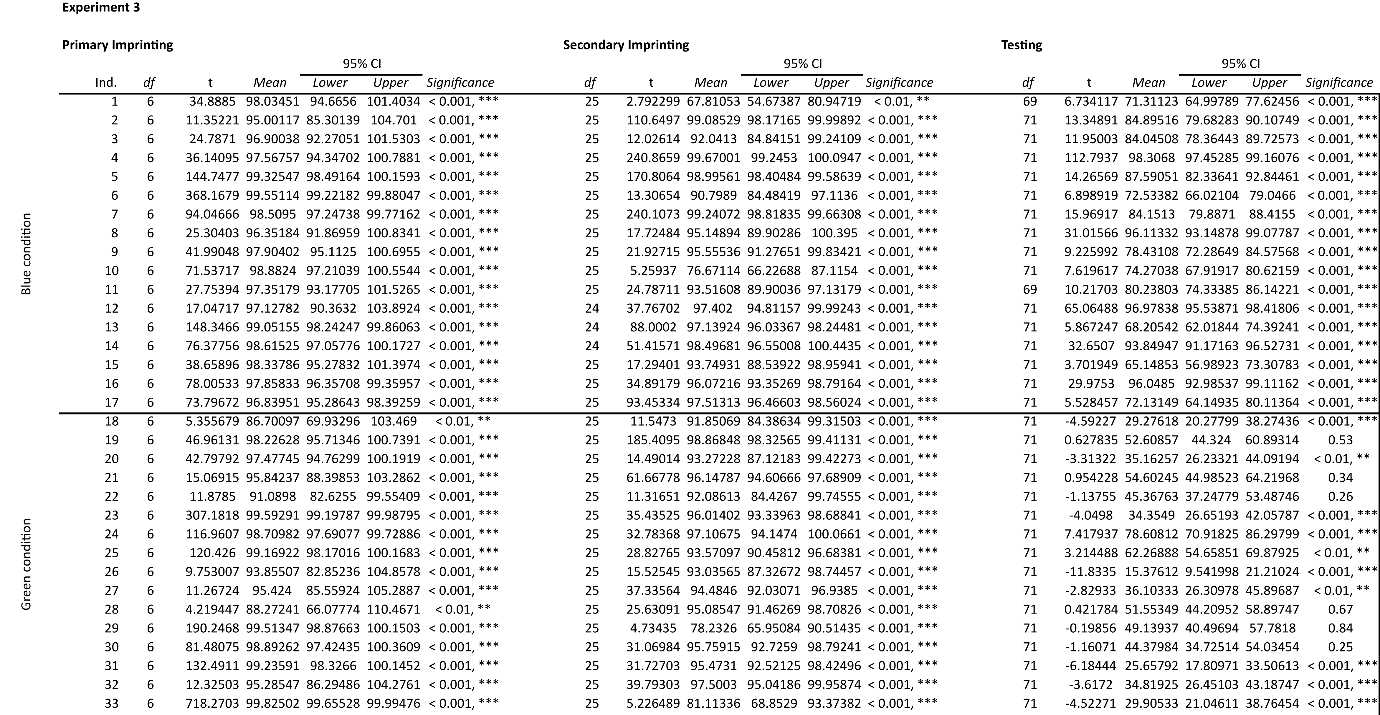


Table 4: Table showing the results of the t-tests on the preference for the primary imprinting stimulus against chance-level (50%) for each individual of the fourth experiment.


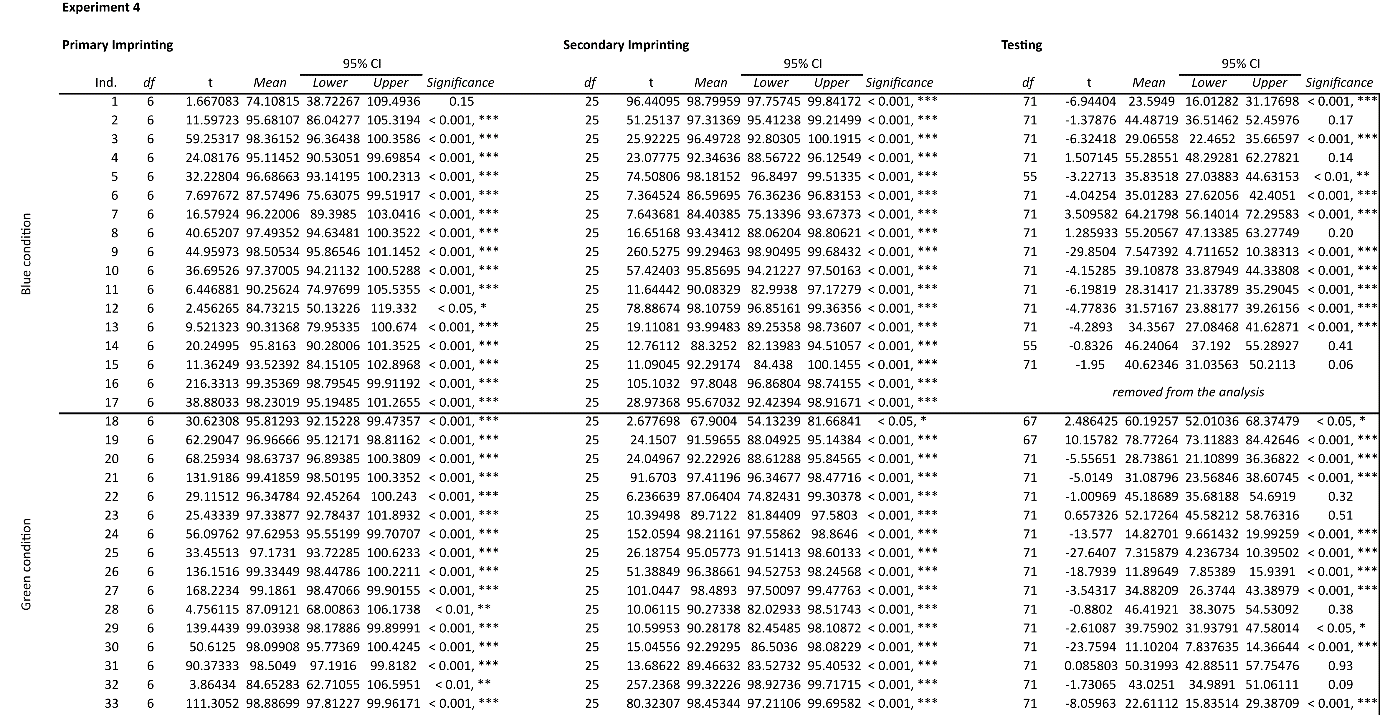

Supplement: Supplementary file 1 — Supplementary Information. [file 41598_2021_86989_MOESM1_ESM.docx]
